# Polymer Analysis and Characterization

**DOI:** 10.3390/polym16243509

**Published:** 2024-12-17

**Authors:** Giulio Malucelli

**Affiliations:** 1 Department of Applied Science and Technology, Viale Teresa Michel 5, 15121 Alessandria, Italy; giulio.malucelli@polito.it; 2 Consorzio Interuniversitario Nazionale per la Scienza e Tecnologia dei Materiali (INSTM), Via G. Giusti 9, 50121 Florence, Italy

**Keywords:** polymer analysis, polymer characterization, polymer nanocomposites, polymer composites, numerical modeling, flame retardancy, structure-property-processing relationships in polymer systems

## Abstract

This editorial aims to summarize some representative research efforts provided by the authors who contributed to the Polymer Analysis and Characterization section of the Polymers journal in the year 2024. The numerous and high-quality research outputs provided so far clearly indicate that the Polymer Analysis and Characterization section of the Polymers journal is rapidly and continuously growing, stimulating more and more researchers to publish their research outcomes here.

Dear colleagues and friends,

As 2024 draws to a close, our Polymer Analysis and Characterization section has enjoyed another fruitful year of high-quality Special Issues and scientific papers (192 published articles to date) on various topics in polymer analysis and characterization. In particular, four main subtopics were considered by the section contributors, namely: the investigation of polymeric nanocomposites, the assessment of the flame-retardant behavior of polymer systems, the design, production, and characterization of polymer composites for advanced applications, and the development of numerical models.

As far as some examples of polymer nanocomposites are considered, Alsoud and co-workers [1] investigated the direct current breakdown characteristics of both unfilled epoxy and epoxy nonconductive nanocomposites (filled with silica, MgO, or alumina). Then, solution casting was selected as a valuable processing method for preparing polyvinyl alcohol-based nanocomposite films incorporating SrTiO_3_ and carbon nanotubes. The obtained systems were found to be suitable for optoelectronic applications [2]. Elhmali and co-workers demonstrated the potential of hybrid nanoparticles made of SrTiO_3_ and MnO_2_ for reinforcing dental poly(methyl methacrylate) [3]; Wang et al. [4] exploited Dynamic Mechanical Analysis for assessing the thermo-mechanical behavior of carbon nanotube/epoxy nanocomposite films. Further, Wu and co-workers [5] succeeded in producing highly thermally conductive, triple-level, ordered, nanofibrous films made of poly(vinyl alcohol) and multi-walled carbon nanotubes, suitable for thermal management applications. The same polymer matrix was investigated by Shui et al. [6], who employed carboxy-functionalized graphene as an effective nanofiller for providing poly(vinyl alcohol) with multifunctional features (namely, with enhanced mechanical, barrier, electrical, and antibacterial properties). Within the current demanding sustainability goals, Uşurelu and co-workers [7] investigated the effect of the incorporation of silanized cellulose nanofibers into Poly(3-hydroxybutyrate) nanocomposites. Cordoba and co-workers [8] exploited an in situ sol–gel synthesis method for obtaining poly(dimethylsiloxane)/silica nanocomposites with tunable properties. Liu and co-workers [9] thoroughly investigated the anti-icing and hydrophobicity performance of nanoparticle/epoxy formulations developed using three types of nanoparticles (namely, ZnO, SiO_2_, and TiO_2_), modified with stearic acid. Nugraha et al. [10] studied the effect of the incorporation of nano-graphite at different loadings on additively manufactured polymer systems obtained through stereolithography and highlighted interesting relationships between the composition of the resin formulations and the properties of the resulting nanocomposites.

As far as the flame-retardant behavior of polymer systems is concerned, Wei and co-workers [11] synthesized a bio-based flame retardant based on phytic acid and chitosan derived from biomass. Then, the so-obtained flame retardant was employed, together with melamine and polyvinyl alcohol, for the design of flame-retardant, intumescent urea/formaldehyde resins, using ammonium polyphosphate and ammonium chloride as curing agents. In a further research effort, Zhang et al. [12] demonstrated that the synergistic combination of ammonium polyphosphate and nickel phytate (synthesized on purpose) was effective in providing enhanced flame-retardant features to rigid polyurethane foams. Alosime and Basfar [13] studied the effect (on both the mechanical behavior and flame retardancy) of the incorporation of carbon nanotubes and carbon black into linear low-density polyethylene/ethylene–vinyl acetate blends containing magnesium hydroxide and huntite hydromagnesite, used as flame retardants. Lu and co-workers [14] exploited a complexation reaction to obtain a self-extinguishing urea-formaldehyde–guanidazole–phytate–copper coating. Boztoprak [15] employed beechwood flour in combination with tetrabromobisphenol-A and antimony trioxide to provide polypropylene with enhanced flame-retardant features, notwithstanding an improvement of such mechanical properties as hardness, impact strength, and wear resistance. Shivakumar and co-workers [16] succeeded in designing and producing fly ash/polyurethane composites with enhanced mechanical, flame-retardant, and dielectric properties.

The Polymer Analysis and Characterization section also benefited from interesting contributions dealing with the design, preparation, and characterization of polymer composites for advanced applications. Some interesting examples are summarized below.

Van Thiem and co-workers [17] incorporated coconut sawdust powder into polypropylene, assessing the effect of the employed compatibilizer (i.e., oleamide) content, the wood powder loading, and the injection molding parameters on the overall mechanical behavior of the resulting composites. Messmer et al. [18] investigated the micro-mechanical behavior of a polyimine-based vitrimer reinforced with basalt fibers as a sustainable alternative to standard composite materials. Song and co-workers [19] studied the effects of three kinds of epoxy resins and their formulating compositions and three ionic types of sizing agents on the interlaminar shear strength and compressive strength of epoxy/carbon fiber composites. Further, a good review paper [20] provided a comprehensive analysis of multiscale defects in fiber-reinforced thermoplastic composites produced via fused filament fabrication, emphasizing the impact of process parameters and the complexities involved in managing these defects. Cheng and co-workers [21] performed low-velocity impact tests and finite element simulation on glass fiber-reinforced polymer hollow-ribbed emergency pipes; the tests were carried out at different impact heights, and the impact response and damage characteristics during impact were evaluated to shed light on the optimal design of these types of structural components. Iquilio et al. [22] explored the mechanical behavior of a vinyl ester polymer matrix reinforced with jute fibers with two different orientations, exploiting tensile tests, digital image correlation techniques, and morphological analyses (i.e., SEM measurements). Shams et al. [23] studied the frontal polymerization of a Bisphenol-A epoxy resin reinforced with either short or continuous carbon fibers. More specifically, the effects of the presence and loading of the reinforcements on the temperature profiles of the exothermic reaction, polymerization frontal velocities, degree of cure, microstructures, and thermal and mechanical properties of the resulting composites were thoroughly investigated. Wang et al. [24] evaluated the durability of two kinds of E-glass fiber-reinforced composites based on an epoxy vinyl ester and an unsaturated polyester matrix and subjected them to hygrothermal aging. Monitoring the mechanical performance during the aging process allowed for comprehensively analyzing the cause of the deterioration of the composites.

Regarding the modeling and simulation research outcomes, Jiang et al. [25] established a 3D helix geometry unit cell to simulate the complex spatial configuration of 3D four-direction carbon/epoxy braided composites. This model allowed for the use of the multiphase finite element method to predict the impacts of environmental temperature on the thermophysical properties of the composites. Verde et al. [26] proposed a numerical model derived from the Tool–Narayanaswamy–Mohynian model to predict the evolution of epoxy’s mechanical properties during the curing process. In addition, the model was implemented in an Ansys APDL environment, assuming a linear viscoelastic behavior. Pursuing this research, the proposed model was then applied to numerically estimate the internal stresses that develop within an epoxy cylinder subjected to a generic thermal history, aiming to evaluate the internal stresses without using experimental techniques and understand the factors influencing them [27]. Tang et al. [28] exploited molecular dynamics simulations to track the network formation and predict the performance of methyl hexahydrophthalic anhydride-cured epoxidized soybean oil/diglycidyl ether of bisphenol-A blends. For this purpose, the composition of the blends (in terms of epoxidized soybean oil content) was successfully correlated with volumetric shrinkage, glass transition temperature, coefficient of thermal expansion, and overall mechanical behavior. Based on the theory of strain energy function, Liu and co-workers [29] proposed a constitutive model considering density and strain rate effects to describe the stress–strain behavior of polyurethane elastomers under various densities and strain rates. The model was found to be in good agreement with the experimental data. Li et al. [30] exploited a method based on the concept of fracture fatigue entropy to simulate the impact of load history on the premature fatigue failure of the viscoelastic polymer matrix in carbon-fiber-reinforced plastics. Compared with the Palmgren–Miner rule, the elaborated model was more reliable and practical for predicting fatigue life under complex loading conditions. Sabol and Murčinková [31] analyzed the stress wave propagation generated by an impulsive unit load in a 2D representative unit cell of a polymer composite embedding circular particles that represent spherical particles, elliptical particles, and short fibers. To this aim, finite element analysis was successfully utilized for the micro-scale numerical simulation.

All the aforementioned research outcomes witness the remarkable research activities carried out in the Polymer Analysis and Characterization section of Polymers.

Further, I would like to remind you that 39 Special Issues and three Topic Collections are still open and ready to receive your valuable manuscripts.

Finally, I hope that, in 2025, the Polymer Analysis and Characterization section will continue to grow and expand with the help of the Editorial Board, the Editorial Staff, and, last but not least, all the esteemed readers and authors!

## Data Availability

Data are contained in the articles cited in the references.
